# Parent-reported sleep problems, symptom ratings, and serum ferritin levels in children with attention-deficit/hyperactivity disorder: a case control study

**DOI:** 10.1186/1471-2431-13-217

**Published:** 2013-12-30

**Authors:** Maha K Abou-Khadra, Omnia R Amin, Olfat G Shaker, Thanaa M Rabah

**Affiliations:** 1 Department of Pediatrics, Faculty of Medicine, Cairo University, 2 Yousef Fahmy Street, El-Areech Street, Al-Ahram Street, Giza, Egypt; 2 Department of Psychiatry, Faculty of Medicine, Cairo University, Cairo, Egypt; 3 Department of Medical Biochemistry and Molecular Biology, Faculty of Medicine, Cairo University, Cairo, Egypt; 4 Department of Community Medicine Research, National Research Centre, Cairo, Egypt

**Keywords:** ADHD, Ferritin levels, Sleep

## Abstract

**Background:**

Sleep problems are common among children with attention-deficit/hyperactivity disorder (ADHD). Serum ferritin levels have been associated with the severity of symptoms and sleep disturbances among children with ADHD. This study was conducted to investigate parent-reported sleep problems in a sample of Egyptian children with ADHD and to examine the relationship between their sleep, symptom-ratings, and low serum ferritin levels.

**Methods:**

Parents of 41 ADHD children, aged 6 to 12 years, filled out the Children’s Sleep Habits Questionnaire (CSHQ) and Conners’ Parent Rating Scale-Revised: Long Version (CPRS-R:L) in Arabic. Serum ferritin levels were determined with an enzyme-linked immunosorbent assay. The parents of the 62 controls filled out the CSHQ.

**Results:**

The ADHD group showed significantly higher scores in CSHQ subscales and total score. Children with serum ferritin levels <30 ng/mL had more disturbed sleep. There were significant negative correlations between sleep duration subscale, total score of CSHQ, and serum ferritin levels. There were no significant differences in hyperactivity, cognitive problems/inattention, oppositional, or ADHD index subscale scores between children with serum ferritin levels <30 ng/mL and those with serum ferritin levels ≥30 ng/mL.

**Conclusions:**

Sleep problems are common, and this study suggests an association between low serum ferritin levels and sleep disturbances.

## Background

Attention-deficit/hyperactivity disorder (ADHD) is a condition characterized by symptoms of inattention or hyperactivity/impulsivity sufficient to cause clinically significant impairment of age-appropriate academic, social, or occupational functioning [1]. Sleep problems have been frequently reported among children with ADHD [2,3]. A recent meta-analysis of subjective and objective studies comparing sleep in children with ADHD versus controls indicated that children with ADHD had significantly higher bedtime resistance, more sleep onset difficulties, night awakenings, difficulties with morning awakenings, sleep-disordered breathing, and daytime sleepiness in subjective studies [4]. Another meta-analysis of relevant polysomnographic studies revealed that children with ADHD are more likely than controls to suffer from periodic limb movements in sleep [5]. A recent review reported that sleep problems are very common in children who have ADHD and that the association between ADHD and sleep disturbances has been relatively overlooked in research conducted on ADHD populations [6]. As recommended by Konofal and colleagues [7], the relationship between sleep disorders and ADHD should be considered by healthcare practitioners as part of the global approach to the management of ADHD. A previous study reported low serum ferritin levels among children with ADHD [8]. Similarly, a recent Egyptian study reported low serum ferritin level among Egyptian children with ADHD [9]. Serum ferritin levels have been associated with the severity of symptoms [10,11]. Although several studies have examined the effect of low serum ferritin levels on the severity of symptoms, few studies have examined their effect on the sleep of children with ADHD [12,13]. The aim of this study was to describe sleep problems in a sample of Egyptian children with ADHD and to examine the relationship between their sleep, symptom ratings, and low serum ferritin levels. We hypothesized that sleep problems are common among Egyptian children with ADHD, and we expect a significant association between sleep disturbances, symptoms severity, and low serum ferritin levels.

## Methods

### Participants

Forty-one non-medicated children with ADHD aged between 6 and 12 years were recruited from a patient population referred to the pediatric psychiatry clinic in the Centre of Preventative Medicine, Cairo University. Children with medical disorders, co-morbid psychiatric disorders or receiving medications known to affect sleep were not recruited. Parents of the ADHD group were given informed consent forms and a brief survey regarding parent’s education and any significant medical problems and/or medication for their child, in addition to the Children’s Sleep Habits Questionnaire (CSHQ) [14] and Conners’ Parent Rating Scale-Revised: Long Version (CPRS-R:L) [15] in Arabic. The Stanford Binet intelligence test was carried out as a routine clinical activity for the diagnosis of ADHD. All children with ADHD were of average or low average IQ according to the Stanford Binet intelligence test. All children with ADHD and their parents were subjected to a semi-structured psychiatric interview by a consultant child psychiatrist, and ADHD was diagnosed according to DSM-IV-TR criteria [1]. Sixty-two control normal healthy children free of ADHD criteria or significant learning disabilities, according to clinical screening were included from among the children of nursing staff and relatives of ADHD children. The parents of the control group filled out the CSHQ and brief survey regarding parent’s education and any significant medical problems and/or medication for their child. For all illiterate parents, the researchers read the questions and provided a full explanation to avoid any misunderstanding and wrong answers. The study was approved by the research committee at the Department of Paediatrics, Faculty of Medicine, Cairo University. Ethical approval was obtained from the Institutional Review Board for Human Subject Research at National Hepatology & Tropical Medicine Research Institute, Cairo, Egypt. Written informed consent was obtained from the parents of children participating in the study.

### Measure of sleep patterns and sleep problems

The abbreviated version of the Children’s Sleep Habits Questionnaire (CSHQ) [14] was used to assess sleep habits and sleep problems as reported by parents. The CSHQ consists of 33 sleep-disturbance items and 3 items asking for information about bedtime, morning waking time, and daily total sleep duration. Parents are asked to recall their child’s sleep behaviors over a “typical” recent week. Each item is rated on a three-point scale: 1 = rarely (0–1 time/week); 2 = sometimes (2–4 times/week); 3 = usually (5–7 times/week). Some items that are considered to be desirable sleep behaviors are reversed in scoring, such that a higher score reflects more disturbed sleep behavior. Needing parent in room to sleep and afraid of sleeping alone items are present in both bedtime resistance and sleep anxiety subscales. The 33 sleep-disturbance items are conceptually grouped into eight subscales: bedtime resistance (6 items), sleep-onset delay (1 item), sleep duration (3 items), sleep anxiety (4 items), night wakings (3 items), parasomnias (7 items), sleep-disordered breathing (3 items), and daytime sleepiness (8 items). With permission of the original author, the CSHQ was translated into Arabic and was used in a previous study in our community [16]. Reliability analysis showed that Cronbach’s alpha was 0.85 for the ADHD group and 0.84 for the control group.

### Conners’ Parent Rating Scale-Revised: Long Version (CPRS-R:L)

Conners’ Parent Rating Scale-Revised: Long Version (CPRS-R:L) was used to assess symptom severity as reported by parents [15]. This form includes 80 items grouped into different subscales: oppositional, cognitive problems/inattention, hyperactivity, anxious-shy, ADHD index, perfectionism, social problems, psychosomatic, Conners’ Global index, and DSM-IV symptoms. The CPRS-R:L in Arabic was developed by translation and back-translation with permission of the original author. Reliability analysis showed that Cronbach’s alpha was 0.95.

### Serum ferritin levels

Serum ferritin concentration was measured using the Ferritin AccuBind ELISA test system from Monobind inc. (USA). Low serum ferritin levels in this study were defined as ferritin < 30 ng/mL, a measure used in a previous study [8].

### Statistical analysis

Data were analyzed using SPSS for Windows statistical package version 17 (SPSS Inc., Chicago, IL). Numerical data were expressed as means and standard deviations. Categorical data were expressed as frequencies and percentages. Significant differences between groups were tested using a chi-square test for categorical variables. Comparison between two groups was performed using an independent sample t-test. Pearson correlation was used to test correlation between sleep, symptoms severity, and ferritin levels. The data subjected to correlation analysis were normally distributed for parametric tests. A p-value < 0.05 was considered significant.

## Results

### Sample characteristics

Of the total 41 child participants, 35 (85.4%) were boys and 6 (14.6%) were girls. Their ages ranged from 6 to 12 years, with a mean age of 8.03 years (SD = 1.66). The numbers of children with T-scores > 70 (markedly atypical) in oppositional, cognitive problems/inattention, hyperactivity, and ADHD index subscales were 32 (78.0%), 31(75.6%), 33 (80.5%), and 20 (48.8%), respectively. In this sample, 15.0% of fathers were illiterate, 65.0% had a high-school education or less, and 20.0% had graduated from university. Regarding the mothers’ level of education, 12.8% were illiterate, 69.2% had a high-school education or less, and 17.9% had graduated from university. The control group consisted of 62 healthy children, 55 of them were relatives of children with ADHD. The mean age of the control group was 8.60 ± 1.87; 43 (70.5%) were boys. There were no significant differences between the ADHD and control groups with respect to age (t = 1.594, p = 0.114), sex (*x*^2^ = 2.980, p = 0.084), fathers’ level of education (*x*^2^ = 1.680, p = 0.314), or mothers’ level of education (*x*^2^ = 2.579, p = 0.303).

### Sleep/wake patterns

The mean (±SD) night bedtime of the ADHD group was 23.15 ± 1.34, the mean morning wake-up time was 8.11 ± 1.42, and the mean total sleep duration was 9.40 ± 1.66 h. The mean (±SD) night bedtime of the control group was 23.24 ± 1.52, the mean morning wake-up time was 7.77 ± 2.23, and the mean total sleep duration was 9.42 ± 1.38 h. There was a significant difference between the ADHD and control groups with respect to bed time (t = 2.220, p = 0.029). There were no significant differences in wake-up time (t = 0.891, p = 0.376) or total sleep duration (t = 0.062, p = 0.951).

### Comparisons of CSHQ scale scores

Comparisons of CSHQ scale scores for the ADHD and control groups are shown in Table 1. There were significant differences in bedtime resistance, sleep anxiety, parasomnias, sleep-disordered breathing, daytime sleepiness, and total scale score (p < 0.05). There was marginal difference between groups in sleep duration subscale (p = 0.056). There were no significant differences with respect to sleep-onset delay or night wakings subscales (p > 0.05).

**Table 1:** Comparison of CSHQ scale scores between ADHD and control groups

	**ADHD**	**Controls**		
	**Mean ± SD(n)**	**Mean ± SD(n)**	**t**	**p**
CSHQ subscales				
Bedtime resistance	11.9 ± 3.0(41)	9.4 ± 2.2(62)	4.906	<0.001
Sleep-onset delay*	1.8 ± 0.7(40)	1.7 ± 0.8(61)	0.294	0.769
Sleep duration*	5.2 ± 1.7(41)	4.6 ± 1.7(61)	1.933	0.056
Sleep anxiety	8.0 ± 2.4(41)	6.1 ± 2.0(62)	4.413	<0.001
Night wakings*	5.1 ± 1.9(40)	5.1 ± 1.5(62)	0.085	0.932
Parasomnias	11.9 ± 2.9(41)	9.6 ± 3.3(62)	3.567	0.001
Sleep-disordered breathing*	4.6 ± 1.9(40)	3.9 ± 1.5(62)	2.021	0.046
Daytime sleepiness	15.9 ± 3.5(41)	14.2 ± 3.3(62)	2.402	0.018
Total score	60.0 ± 10.4(41)	51.5 ± 9.2(62)	4.400	<0.001

*Numbers of subjects do not add up to total N because of missing data.

### The effect of ferritin levels on CSHQ scale scores

As shown in Table 2, there were no significant differences in CSHQ subscale scores or the total score with respect to ferritin levels (p > 0.05). There was a marginal difference between groups with respect to the total score (p = 0.05).

**Table 2:** The Children’s Sleep Habits Questionnaire scale scores according to ferritin levels

	**<30 ng/mL**	**≥30 ng/mL**		
	**Mean ± SD(n)**	**Mean ± SD(n)**	**t**	**p**
CSHQ subscales				
Bedtime resistance	12.3 ± 2.9(25)	11.3 ± 3.0(16)	1.093	0.281
Sleep-onset delay*	1.9 ± 0.7(24)	1.6 ± 0.7(16)	1.316	0.196
Sleep duration	5.6 ± 1.7(25)	4.7 ± 1.5(16)	1.750	0.088
Sleep anxiety	8.4 ± 1.9(25)	7.4 ± 3.0(16)	1.133	0.269
Night wakings*	5.5 ± 1.5(24)	4.6 ± 2.3(16)	1.398	0.170
Parasomnias	12.1 ± 3.1(25)	11.6 ± 2.5(16)	0.556	0.581
Sleep-disordered breathing*	5.0 ± 2.2(25)	4.1 ± 1.2(15)	1.703	0.097
Daytime sleepiness	16.7 ± 2.5(25)	14.6 ± 4.5(16)	1.714	0.101
Total score	62.5 ± 8.5(25)	56.1 ± 12.0(16)	2.00	0.052

*Numbers of subjects do not add up to total N because of missing data.

### Correlation between serum ferritin levels and CSHQ scale scores

As shown in Table 3, there were significant negative correlations between serum ferritin levels and sleep duration subscale (*r =* -0.309, p = 0.049) and total score (*r =* -0.363, p = 0.020).

**Table 3:** Correlation between serum ferritin levels and CSHQ scale scores

	***r***	**p**
CSHQ subscales		
Bedtime resistance	-0.240	0.131
Sleep-onset delay	-0.239	0.137
Sleep duration	-0.309	0.049
Sleep anxiety	-0.284	0.072
Night wakings	-0.289	0.070
Parasomnias	-0.139	0.388
Sleep-disordered breathing	-0.195	0.227
Daytime sleepiness	-0.286	0.070
Total score	-0.363	0.020

### The effect of ferritin levels on CPRS-R:L subscale scores

As shown in Table 4, there were no significant differences in hyperactivity, cognitive problems/inattention, oppositional, or ADHD index subscale scores between children with serum ferritin levels <30 ng/mL and those with serum ferritin levels ≥30 ng/mL (p > 0.05).

**Table 4:** Conners’ Parent Rating Scale-Revised: Long Version (CPRS-R:L) subscales according to ferritin levels

	**<30 ng/mL**	**≥30 ng/mL**		
	**Mean ± SD**	**Mean ± SD**	**t**	**p**
	**(n = 25)**	**(n = 16)**		
CPRS-R:L subscales				
Oppositional	21.2 ± 4.0	18.4 ± 5.9	1.761	0.086
Cognitive Problems/Inattention	26.6 ± 6.7	26.4 ± 5.5	0.132	0.896
Hyperactivity	18.6 ± 4.8	16.4 ± 4.4	1.530	0.134
ADHD index	26.7 ± 5.5	24.7 ± 4.3	1.250	0.219

### Correlation between serum ferritin levels and CPRS-R:L subscale scores

Table 5 shows that there were no significant correlations between serum ferritin levels and hyperactivity, cognitive problems/inattention, oppositional, or ADHD index subscale scores (p > 0.05).

**Table 5:** Correlation between serum ferritin levels and Conners’ Parent Rating Scale-Revised: Long Version (CPRS-R:L) subscale raw scores

	***r***	**p**
CPRS-R:L subscales		
Oppositional	-0.277	0.080
Cognitive Problems/Inattention	-0.044	0.784
Hyperactivity	-0.094	0.559
ADHD index	-0.003	0.984

## Discussion

We conducted this study to describe sleep problems in a sample of Egyptian children with ADHD and to investigate the relationship between their sleep, symptom-ratings, and low serum ferritin levels. The results show that the ADHD group had significantly higher scores in bedtime resistance, sleep anxiety, parasomnias, sleep-disordered breathing, daytime sleepiness, and global sleep disturbance (CSHQ total score) than the control group. Our results are in agreement with those of previous studies that have reported a high prevalence of sleep disturbances in children with ADHD. Owens et al. [17] found that children with ADHD had significantly higher scores on all sleep subscales of the CSHQ than did controls. A recent study of 27 children with ADHD and 26 healthy controls reported that the ADHD group had significantly higher scores with respect to sleep-onset delay, sleep duration, night waking, parasomnias, daytime sleepiness, and total sleep disturbance factors [18]. As reviewed by Owens [19], several recent reports have documented a significant increase in parent-reported sleep-disordered breathing symptoms specifically in children being evaluated for or diagnosed with ADHD. Golan and colleagues [20] reported that 50% of children that were diagnosed with ADHD had signs of sleep-disordered breathing, compared with 22% of children in the control group. In agreement with these studies, our study revealed higher sleep-disordered breathing subscale scores in children with ADHD. Several studies using the multiple sleep latency test have revealed that children with ADHD exhibit significantly more daytime sleepiness than controls [20,21]. A recent systematic review suggested that children with ADHD exhibit a greater extent of daytime sleepiness than controls [22]. Recently, an association between obesity and ADHD has been reported [23], and excessive daytime sleepiness has been implicated in this association [24]. The present study showed that children with ADHD had higher daytime sleepiness subscale scores than did controls. Gruber and colleagues [25] reported an ADHD group with higher scores regarding insufficient sleep and sleep anxiety factors, and they suggested that sleep-onset problems and daytime sleepiness in children with ADHD resemble the clinical picture of circadian phase delay and that the sleep problems that characterized children with ADHD might be related to the circadian system. Van der Heijden and colleagues [26] reported that children with ADHD and chronic idiopathic sleep-onset insomnia showed a delayed sleep phase and delayed dim-light melatonin onset compared with ADHD children without sleep-onset insomnia. This proposed circadian rhythm model could explain the high rate of bedtime resistance and daytime sleepiness among our sample.

Another important finding of this study is the association between serum ferritin levels and sleep disturbances. Previous studies have demonstrated a significant relation between serum ferritin levels and sleep disturbances in children with ADHD. Konofal et al. [27] reported improvement in sleep problems, assessed by parental interview and sleep diaries filled out by the parents, in a child with ADHD after iron supplementation for low ferritin levels. Another study reported improvement in total score, hyperactive/impulsive, and inattentive subscales of the ADHD Rating Scale and restless leg symptoms after iron supplementation [28]. In a recent study of 68 children with ADHD, aged 6 to 14 years, Cortese and colleagues [13] found that children with serum ferritin levels <45 μg/L had significantly higher scores on the sleep-wake transition disorders (SWTD) subscale of the Sleep Disturbance Scale for Children (SDSC) compared with children with serum ferritin levels ≥ 45 μg/L, and there was a significant inverse correlation between the serum ferritin levels and SWTD scores. The authors concluded that serum ferritin levels <45 μg/L might indicate a risk for sleep-wake transition disorders, including abnormal sleep movements, in children with ADHD. In agreement with these studies, our study revealed a significant negative correlation between serum ferritin levels and global sleep disturbance score (CSHQ total score). In addition, children with low serum ferritin had higher scores on CSHQ scales. However, this difference did not reach statistical significance, which could be attributed to the small sample size of this study. A future study featuring a larger sample size might reveal significant differences.

The mechanism through which low serum ferritin levels could affect the sleep of children with ADHD has not been previously studied. A recent study of brain iron levels in children with and without ADHD using magnetic resonance imaging reported that children with ADHD showed significantly lower estimated brain iron levels in the right and left thalamus compared to healthy controls and suggested that low iron in the thalamus may contribute to ADHD pathophysiology [29]. Konofal and colleagues [28] suggested that brain iron stores influence the monoamine-dependent functions in ADHD. The catecholamine systems have been implicated in the regulation of sleep and arousal [6]. It was proposed that a potential dysfunction of dopaminergic pathways may play a significant role in the association between iron deficiency and increased motor activity in the sleep of children with ADHD [13].

A number of studies have demonstrated a significant association between serum ferritin levels and severity of symptoms in children with ADHD [10,11]. A recent Indian study [30] revealed a significant negative correlation between serum ferritin levels and oppositional sub-score on Conners’ Rating Scale. Oner and colleagues [10] reported that lower ferritin levels were associated with higher hyperactivity scores in parental ratings. In contrast to previous studies, Millichap et al. [31] found no significant difference in severity of attention-deficit hyperactivity disorder symptoms in 12 children with serum ferritin levels <20 ng/mL compared with 12 children with levels >60 ng/mL. Another recent study assessed the serum ferritin levels in 101 children with ADHD and 93 controls and examined the association between serum ferritin levels and ADHD severity, reporting that serum ferritin levels did not significantly differ between children with ADHD and controls and correlations between serum ferritin levels and measures related to ADHD severity were not significant [32]. Similarly, we did not find significant correlations between serum ferritin levels and symptoms severity. Moreover, there were no significant differences in hyperactivity, cognitive problems/inattention, oppositional, or ADHD index subscales with respect to serum ferritin levels.

The main limitation of this study is that children’s sleep and symptom ratings were assessed by reports from parents, who were the only informants, which may lead to over-or underestimation of sleep and symptom severity. Another limitation is that children were recruited from a psychiatric clinic that may attract severe cases of ADHD; thus, the results may not be generalized to community children with ADHD. The sample size of this study was small and further research with a larger sample size is recommended. An objective study using polysomnography would be more effective in examining sleep disturbances among children with ADHD rather than parental reports, and further research is required to investigate the impact of low serum ferritin levels on children’s sleep and to understand the possible mechanisms underlying their behavior.

## Conclusions

Sleep problems are common among this sample of children with ADHD. This study suggests an association between low serum ferritin levels and sleep disturbances among Egyptian children with ADHD.

## Abbreviations

ADHD: Attention deficit hyperactivity disorder; CSHQ: Children’s sleep habits questionnaire; CPRS-R: L: Conners’ parent rating scale-revised: Long version; SWTD: Sleep-wake transition disorders; SDSC: Sleep disturbance scale for children.

## Competing interests

The authors declare that they have no competing interests.

## Authors’ contributions

MKA and ORA contributed to the conception and design of this study. OGS performed the laboratory analysis, and TMR performed the statistical analysis. All authors read and approved the final manuscript.

## Pre-publication history

The pre-publication history for this paper can be accessed here:

http://www.biomedcentral.com/1471-2431/13/217/prepub
